# Literary Notices

**Published:** 1877-02-20

**Authors:** 


### LITERARY NOTICES.

*Lippincott's Magazine.*—The January and February numbers of this beautiful magazine is on our table. They are filled with handsome illustrations of foreign and home scenes taken from nature. The literature is attractive—coming from the pens of the best of American and English authors. The " Marquis of Lossie," by Rev. Geo. McDonald, is a serial story of great power, and is worth the price of subscription—$4.00.

*Appleton's Journal.*—This delightful home journal—once a weekly, now a monthly—is one of the very best of our literary magazines. The illustrations are handsome and the stories and other reading, are first class. It will be a family friend and a genial monthly visitor in the home circle, and one cheaply secured at the low price of $4.00.

*The Scientific American*, and the *Scientific American Supplement*, are journals our readers should take. They are truly scientific—covering a wide field in science, literature and art. Munn & Co., New York.

Vick's garden and floral seeds are the finest and best we have ever used. We have used his seeds for several years and they are pure—just what they are recommended to be. Get Vick's seed. Address James Vick. Rochester, New York. G.

